# Publisher Correction: Differences in age-specific mortality between wild-caught and captive-born Asian elephants

**DOI:** 10.1038/s41467-018-06032-4

**Published:** 2018-08-28

**Authors:** Mirkka Lahdenperä, Khyne U. Mar, Alexandre Courtiol, Virpi Lummaa

**Affiliations:** 1 Department of Biology, University of Turku, 20014 Turku, Finland; 2 Department of Evolutionary Genetics, Leibniz Institute for Zoo and Wildlife Research, Alfred-Kowalke-Strasse, Berlin 10315, Germany

Correction to: *Nature Communications*; doi:10.1038/s41467-018-05515-8; published online: 07 Aug 2018

The original version of this Article incorrectly cited as ref. 45 the paper ‘Lynch, E. C., Lahdenperä, M., Mar, K. U. & Lummaa, V. The evolutionary significance of maternal kinship in a long-lived mammal. *Philos. Trans. R. Soc. Lond. B* (2018, in press)’ in the eighth sentence of the last paragraph of the Introduction, and the last sentence of the first paragraph of the ‘Study population’ section of the Methods. As this paper does not exist, this reference has been removed and all following citations have been renumbered as appropriate. This has been corrected in both the PDF and HTML versions of the Article.

