# Supplementary material for: Differences in age-specific mortality between wild-caught and captive-born Asian elephants
Source: Nat Commun. 2018 Aug 7;9:3023. doi: 10.1038/s41467-018-05515-8 (PMC6081384; doi:10.1038/s41467-018-05515-8)
Supplement: Supplementary file 1 — Supplementary Information [file 41467_2018_5515_MOESM1_ESM.pdf]

Differences in age-specific mortality between wild-caught and captive-born Asian elephants

Lahdenperä et al.

Supplementary Table 1. Model averaged estimates and 95% confidence intervals for all parameters. Columns provide, from left to right, the names of the parameters, the model averaged estimate, the model averaged standard error, the lower boundary of the 95% CI based on the model averaged standard error, and the corresponding upper boundary. The last columns visually indicates cases for which the model averaged lower boundary of the 95% is larger than 1e-5.

Parameter	Mean	SE	Lwr	Upr	
w1.females	0.0807	0.00549	0.06994	0.09145	*
w1.males	0.10465	0.00655	0.09182	0.11748	*
b1.females	0.34585	0.03136	0.28439	0.40731	*
b1.males	0.30425	0.02251	0.26013	0.34836	*
w2.females	0.00101	0.00031	0.00041	0.0016	*
w2.males	0.0027	0.0006	0.00152	0.00388	*
b2.females	0.07656	0.00717	0.0625	0.09062	*
b2.males	0.06513	0.00564	0.05408	0.07618	*
w3.females	0	0	0	0	
w3.males	0	0	0	0	
w3.2000	0.00426	0.00156	0.0012	0.00731	*
w3.1940	0.00332	0.00334	-0.0032	0.00987	
w3.1960	8.3E-89	1.9E-20	-4E-20	3.7E-20	
w3.1980	0.00211	0.00099	0.00017	0.00404	*
w3.Unknown	0.00834	0.00266	0.00313	0.01355	*
w3.Ayeyarwa	1.3E-42	0.00158	-0.0031	0.00309	
w3.Bago	7.9E-43	0.00049	-0.001	0.00097	
w3.Kachin	0.00231	0.00168	-0.001	0.00561	
w3.Magway	1.6E-89	0.00056	-0.0011	0.00109	
w3.Mandalay	0.00682	0.00164	0.0036	0.01004	*
w3.Rakhine	2.7E-45	0.00022	-0.0004	0.00043	
w3.Sagaing	0.00147	0.00111	-0.0007	0.00364	
w3.Shan	0.00617	0.00208	0.0021	0.01024	*
w3.Tanintha	0.00354	0.00469	-0.0057	0.01274	
w4.females_IMM	0.02209	0.01318	-0.0037	0.04792	
w4.males_IMM	0.02238	0.01386	-0.0048	0.04955	
w4.females_MILARSHI	0.03286	0.02038	-0.0071	0.07281	
w4.males_MILARSHI	0.03316	0.02068	-0.0074	0.07368	
w4.females_STOCKADE	0.02268	0.01121	0.00072	0.04464	*
w4.males_STOCKADE	0.02298	0.01198	-0.0005	0.04646	
w5.females_IMM	0.0011	0.0007	-0.0003	0.00247	
w5.males_IMM	0.00112	0.00072	-0.0003	0.00253	
w5.females_MILARSHI	0.00085	0.00088	-0.0009	0.00258	
w5.males_MILARSHI	0.00088	0.00091	-0.0009	0.00265	
w5.females_STOCKADE	0.00076	0.00052	-0.0003	0.00177	
w5.males_STOCKADE	0.00078	0.00056	-0.0003	0.00188	
b4.females_IMM	0.34127	0.12858	0.08926	0.59328	*
b4.males_IMM	0.3413	0.16103	0.02568	0.65693	*
b4.females_MILARSHI	0.55583	0.47577	-0.3766	1.48832	
b4.males_MILARSHI	0.55587	0.48553	-0.3958	1.50749	
b4.females_STOCKADE	0.33394	0.24201	-0.1404	0.80828	
b4.males_STOCKADE	0.33398	0.26071	-0.177	0.84496	
b5.females_IMM	0.00622	0.04857	-0.089	0.10142	
b5.males_IMM	0.00613	0.05043	-0.0927	0.10497	
b5.females_MILARSHI	0.0059	0.0486	-0.0894	0.10115	
b5.males_MILARSHI	0.00581	0.05045	-0.0931	0.10469	
b5.females_STOCKADE	0.006	0.04979	-0.0916	0.10358	
b5.males_STOCKADE	0.00591	0.0516	-0.0952	0.10704	

Supplementary Table 2. Estimates for all 17 model fits and for the model averaging. The first column provides the names of the parameters. The rest of the table provides the estimated parameter values for all parameters and for all fitted models. Parameters have been labeled according to the model definition provided in methods. Estimates at zero can either correspond to model constraints or to parameter estimated at such values (see Table 1).

Parameter	mod1	mod2	mod3	mod4	mod5	mod6	mod7	mod8	mod9	mod10	mod11	mod12	mod13	mod14	mod15	mod16	mod17
w1.females	0.08067	0.08067	0.0809	0.0809	0.08051	0.08051	0.08082	0.08082	0.0807	0.0807	0.08089	0.08089	0.07752	0.08862	0.08486	0	0
w1.males	0.10471	0.10471	0.10484	0.10484	0.10492	0.10492	0.10469	0.10469	0.10458	0.10458	0.10465	0.10464	0.10196	0.08862	0.08486	0	0
b1.females	0.34394	0.34393	0.35055	0.35055	0.34376	0.34376	0.35098	0.35098	0.34607	0.34607	0.35189	0.35189	0.29826	0.29176	0.37906	0	0
b1.males	0.30314	0.30314	0.30857	0.30857	0.30571	0.30571	0.31049	0.31049	0.30379	0.30379	0.30862	0.30862	0.27256	0.29176	0.37906	0	0
w2.females	0.00095	0.00095	0.0011	0.0011	0.00103	0.00103	0.00117	0.00117	0.00101	0.00101	0.00116	0.00116	0.002	0.00257	0	0.0053	0
w2.males	0.00261	0.00261	0.0028	0.0028	0.00258	0.00258	0.00293	0.00293	0.00273	0.00273	0.00292	0.00292	0.00385	0.00257	0	0.0053	0
b2.females	0.07733	0.07733	0.07478	0.07478	0.07624	0.07624	0.07375	0.07375	0.07661	0.07661	0.07392	0.07392	0.06069	0.0576	0	0	0
b2.males	0.06561	0.06561	0.06454	0.06454	0.06605	0.06605	0.06368	0.06368	0.06496	0.06496	0.06376	0.06376	0.05614	0.0576	0	0	0
w3.females	0	0	0	0	0	0	0	0	0	0	0	0	0	0	0	0	0
w3.males	0	0	0	0	0	0	0	0	0	0	0	0	0	0	0	0	0
w3.2000	0.00415	0.00415	0.00417	0.00417	0.00433	0.00433	0.00434	0.00434	0.00432	0.00432	0.00432	0.00432	0.0009	0.00197	0.00737	0.02688	0.0292
w3.1940	0.00358	0.00358	0.0056	0.0056	0.00284	0.00284	0.00538	0.00538	0.00277	0.00277	0.00522	0.00522	0.01236	0.01064	0.03445	0.02798	0.0303
w3.1960	0	0	0	0	0	0	0	0	0	0	0	0	0	0	0.01139	0.00611	0.00843
w3.1980	0.00198	0.00198	0.0018	0.0018	0.00223	0.00223	0.00197	0.00197	0.00222	0.00222	0.00198	0.00198	0.00077	0.00146	0.00741	0.00989	0.01221
w3.Unknown	0.00853	0.00853	0.00816	0.00816	0.00839	0.00839	0.00797	0.00797	0.00828	0.00828	0.00795	0.00795	0.00942	0.01185	0.01438	0.01762	0.0206
w3.Ayeyarwa	0	0	0	0	0	0	0	0	0	0	0	0	0.00302	0.00399	0.00846	0.00211	0.00508
w3.Bago	0	0	0	0	0	0	0	0	0	0	0	0	0.00183	0.00241	0.00825	0.00709	0.01007
w3.Kachin	0.00286	0.00286	0.00274	0.00274	0.00203	0.00203	0.00193	0.00193	0.00201	0.00201	0.00193	0.00193	0.00561	0.00602	0.01052	0.01046	0.01344
w3.Magway	0	0	0	0	0	0	0	0	0	0	0	0	0	0	0.00213	0.00417	0.00715
w3.Mandalay	0.00682	0.00682	0.0068	0.0068	0.00685	0.00685	0.00681	0.00681	0.00682	0.00682	0.0068	0.0068	0.00771	0.00787	0.01209	0.01634	0.01932
w3.Rakhine	0	0	0	0	0	0	0	0	0	0	0	0	6.31E-06	0.00021	0.00434	0.00165	0.00463
w3.Sagaing	0.00162	0.00162	0.00147	0.00147	0.00141	0.00141	0.00131	0.00131	0.0014	0.0014	0.0013	0.0013	0.00241	0.00259	0.00636	0.00616	0.00914
w3.Shan	0.00631	0.00631	0.0061	0.0061	0.00615	0.00615	0.00601	0.00601	0.00612	0.00612	0.00602	0.00602	0.00933	0.00959	0.0146	0.01732	0.0203
w3.Tanintha	0.00376	0.00376	0.00404	0.00404	0.0034	0.0034	0.00382	0.00382	0.00334	0.00334	0.00375	0.00375	0.00942	0.00902	0.013	0.01323	0.01621
w4.females_IMM	0.01552	0.01551	0.04325	0.04325	0.01909	0.01909	0.03598	0.03598	0.02243	0.02243	0.0383	0.0383	0	0	0	0	0
w4.males_IMM	0.01552	0.01551	0.04325	0.04325	0.0233	0.02329	0.04367	0.04367	0.02243	0.02243	0.0383	0.0383	0	0	0	0	0
w4.females_MILARSHI	0.04737	0.04736	0.05886	0.05886	0.01909	0.01909	0.03598	0.03598	0.02243	0.02243	0.0383	0.0383	0	0	0	0	0
w4.males_MILARSHI	0.04737	0.04736	0.05886	0.05886	0.0233	0.02329	0.04367	0.04367	0.02243	0.02243	0.0383	0.0383	0	0	0	0	0
w4.females_STOCKADE	0.01939	0.01939	0.03079	0.03079	0.01909	0.01909	0.03598	0.03598	0.02243	0.02243	0.0383	0.0383	0	0	0	0	0
w4.males_STOCKADE	0.01939	0.01939	0.03079	0.03079	0.0233	0.02329	0.04367	0.04367	0.02243	0.02243	0.0383	0.0383	0	0	0	0	0
w5.females_IMM	0.00173	0.00173	0	0	0.00098	0.00098	0	0	0.00097	0.00097	0	0	0	0	0	0	0
w5.males_IMM	0.00173	0.00173	0	0	0.00139	0.00139	0	0	0.00097	0.00097	0	0	0	0	0	0	0
w5.females_MILARSHI	0.00094	0.00094	0	0	0.00098	0.00098	0	0	0.00097	0.00097	0	0	0	0	0	0	0
w5.males_MILARSHI	0.00094	0.00094	0	0	0.00139	0.00139	0	0	0.00097	0.00097	0	0	0	0	0	0	0
w5.females_STOCKADE	0.00065	0.00065	0	0	0.00098	0.00098	0	0	0.00097	0.00097	0	0	0	0	0	0	0
w5.males_STOCKADE	0.00065	0.00065	0	0	0.00139	0.00139	0	0	0.00097	0.00097	0	0	0	0	0	0	0
b4.females_IMM	0.29329	0.29329	0.34459	0.34461	0.35565	0.35565	0.39176	0.39175	0.36111	0.36111	0.40387	0.40384	0	0	0	0	0
b4.males_IMM	0.29329	0.29329	0.34459	0.34461	0.34864	0.34856	0.44172	0.44169	0.36111	0.36111	0.40387	0.40384	0	0	0	0	0
b4.females_MILARSHI	0.87916	0.87905	0.96068	0.96074	0.35565	0.35565	0.39176	0.39175	0.36111	0.36111	0.40387	0.40384	0	0	0	0	0
b4.males_MILARSHI	0.87916	0.87905	0.96068	0.96074	0.34864	0.34856	0.44172	0.44169	0.36111	0.36111	0.40387	0.40384	0	0	0	0	0
b4.females_STOCKADE	0.27566	0.27566	0.30857	0.30858	0.35565	0.35565	0.39176	0.39175	0.36111	0.36111	0.40387	0.40384	0	0	0	0	0
b4.males_STOCKADE	0.27566	0.27566	0.30857	0.30858	0.34864	0.34856	0.44172	0.44169	0.36111	0.36111	0.40387	0.40384	0	0	0	0	0
b5.females_IMM	0.03569	0	0	0.01912	0.01918	0	0	5.54E-05	0.02698	0	0	0.12071	0	0	0	0	0
b5.males_IMM	0.03569	0	0	0.01912	5.89E-05	0	0	0.03663	0.02698	0	0	0.12071	0	0	0	0	0
b5.females_MILARSHI	0.00407	0	0	0.08014	0.01918	0	0	5.54E-05	0.02698	0	0	0.12071	0	0	0	0	0
b5.males_MILARSHI	0.00407	0	0	0.08014	5.89E-05	0	0	0.03663	0.02698	0	0	0.12071	0	0	0	0	0
b5.females_STOCKADE	0.02193	0	0	0.01295	0.01918	0	0	5.54E-05	0.02698	0	0	0.12071	0	0	0	0	0
b5.males_STOCKADE	0.02193	0	0	0.01295	5.89E-05	0	0	0.03663	0.02698	0	0	0.12071	0	0	0	0	0

Supplementary Table 3. Standard Errors associated to the estimates presented in Supplementary Table 2.

Parameter	mod1	mod2	mod3	mod4	mod5	mod6	mod7	mod8	mod9	mod10	mod11	mod12	mod13	mod14	mod15	mod16	mod17
w1.females	0.00534	0.00556	0.00538	0.00566	0.00506	0.00617	0.00607	0.0062	0.00566	0.0051	0.00717	0.0044	0.00528	0.00432	0.00384	0	0
w1.males	0.0075	0.00682	0.00624	0.00575	0.00646	0.00652	0.00606	0.00721	0.00722	0.00613	0.00591	0.00633	0.00574	0.00432	0.00384	0	0
b1.females	0.02783	0.0291	0.03612	0.02627	0.02951	0.03329	0.02781	0.02843	0.02864	0.03308	0.0343	0.02397	0.03215	0.0174	0.03234	0	0
b1.males	0.02379	0.01899	0.02268	0.02004	0.02538	0.0226	0.02312	0.02394	0.02703	0.02309	0.02216	0.0186	0.01877	0.0174	0.03234	0	0
w2.females	0.00029	0.00032	0.00033	0.00036	0.00033	0.0003	0.0003	0.00031	0.0003	0.00028	0.00025	0.00027	0.00054	0.00051	0	0.00424	0
w2.males	0.00049	0.00049	0.00065	0.00055	0.00092	0.0007	0.00077	0.00074	0.00073	0.00059	0.00064	0.00048	0.0011	0.00051	0	0.00424	0
b2.females	0.00677	0.00782	0.00673	0.00733	0.0077	0.00762	0.00606	0.00622	0.00686	0.00678	0.0058	0.00631	0.00767	0.00538	0	0.04953	0
b2.males	0.00468	0.00563	0.00595	0.00582	0.00834	0.00668	0.00804	0.00713	0.00728	0.00449	0.00609	0.00434	0.00798	0.00538	0	0.04953	0
w3.females	0	0	0	0	0	0	0	0	0	0	0	0	0	0	0	0	0
w3.males	0	0	0	0	0	0	0	0	0	0	0	0	0	0	0	0	0
w3.2000	0.00155	0.00168	0.0017	0.00151	0.00124	0.00151	0.0016	0.00206	0.00154	0.00138	0.00193	0.00192	0.00149	0.00151	0.00367	0.00369	0.00216
w3.1940	0.00358	0.00344	0.00389	0.00402	0.00353	0.00308	0.00397	0.00376	0.00325	0.00284	0.00397	0.00324	0.00392	0.00348	0.00429	0.00568	0.00424
w3.1960	0	0	0	0	0	0	0	0	5.12E-20	0	0	0	0.00034	2.68E-05	0.00302	0.00244	0.00085
w3.1980	0.00089	0.00106	0.00094	0.00103	0.00104	0.00112	0.0011	0.00082	0.00104	0.00087	0.00098	0.00088	0.00091	0.00102	0.00291	0.00348	0.00125
w3.Unknown	0.00278	0.00244	0.00244	0.00244	0.00246	0.0024	0.0022	0.00194	0.00262	0.00296	0.00197	0.00231	0.00248	0.0025	0.0034	0.00276	0.00248
w3.Ayeyarwa	0.00127	0.00198	0.00117	0.00092	0.00181	0.0011	0.00091	0.00099	0.00079	0.00158	0.00131	0.00139	0.00317	0.00373	0.00375	0.00374	0.00458
w3.Bago	0.00039	0.00033	0.00089	0.00052	0.00046	0.00065	0.00096	0.00062	0.00059	0.00043	0.00052	0.00087	0.00196	0.00187	0.00363	0.00351	0.00208
w3.Kachin	0.00168	0.00174	0.00143	0.0015	0.00159	0.00154	0.00161	0.0017	0.00159	0.00164	0.00119	0.00145	0.00206	0.00178	0.00321	0.00275	0.00186
w3.Magway	0.00072	0.00069	0.00075	0.00044	0.00081	0.00023	0.00029	0.00056	0.00043	0.00048	0.00044	0.00064	0.00085	0.00071	0.003	0.00276	0.00114
w3.Mandalay	0.00167	0.00162	0.00189	0.00166	0.00158	0.0016	0.00158	0.00167	0.00175	0.00158	0.00156	0.00217	0.00231	0.00203	0.00362	0.00312	0.0018
w3.Rakhine	6.17E-05	8.95E-05	0.00054	0.00014	0.00031	0.00016	0.00042	0.0005	0.00019	9.37E-05	0.00056	0.00079	0.00147	0.00185	0.00432	0.00294	0.00256
w3.Sagaing	0.0009	0.00095	0.00117	0.00108	0.00104	0.00114	0.00116	0.00093	0.00091	0.00129	0.00096	0.00094	0.00148	0.00155	0.0032	0.00246	0.00126
w3.Shan	0.00268	0.00233	0.00167	0.00183	0.00186	0.00176	0.00191	0.0019	0.00175	0.00206	0.00201	0.0017	0.00191	0.00219	0.00374	0.00336	0.00243
w3.Tanintha	0.00445	0.0049	0.00496	0.00526	0.00463	0.00484	0.00405	0.00439	0.00411	0.00459	0.00522	0.00557	0.00829	0.00528	0.00544	0.00654	0.00645
w4.females_IMM	0.01346	0.01403	0.00847	0.00902	0.00967	0.00994	0.00532	0.00473	0.00836	0.01017	0.00557	0.00564	0	0	0	0	0
w4.males_IMM	0.01346	0.01403	0.00847	0.00902	0.01992	0.01961	0.01017	0.01482	0.00836	0.01017	0.00557	0.00564	0	0	0	0	0
w4.females_MILARSHI	0.02344	0.02349	0.01883	0.02009	0.00967	0.00994	0.00532	0.00473	0.00836	0.01017	0.00557	0.00564	0	0	0	0	0
w4.males_MILARSHI	0.02344	0.02349	0.01883	0.02009	0.01992	0.01961	0.01017	0.01482	0.00836	0.01017	0.00557	0.00564	0	0	0	0	0
w4.females_STOCKADE	0.01019	0.01195	0.00759	0.00939	0.00967	0.00994	0.00532	0.00473	0.00836	0.01017	0.00557	0.00564	0	0	0	0	0
w4.males_STOCKADE	0.01019	0.01195	0.00759	0.00939	0.01992	0.01961	0.01017	0.01482	0.00836	0.01017	0.00557	0.00564	0	0	0	0	0
w5.females_IMM	0.00069	0.00058	0	0	0.00049	0.00046	0	0	0.00045	0.00043	0	0	0	0	0	0	0
w5.males_IMM	0.00069	0.00058	0	0	0.00084	0.00085	0	0	0.00045	0.00043	0	0	0	0	0	0	0
w5.females_MILARSHI	0.00121	0.00136	0	0	0.00049	0.00046	0	0	0.00045	0.00043	0	0	0	0	0	0	0
w5.males_MILARSHI	0.00121	0.00136	0	0	0.00084	0.00085	0	0	0.00045	0.00043	0	0	0	0	0	0	0
w5.females_STOCKADE	0.00043	0.00045	0	0	0.00049	0.00046	0	0	0.00045	0.00043	0	0	0	0	0	0	0
w5.males_STOCKADE	0.00043	0.00045	0	0	0.00084	0.00085	0	0	0.00045	0.00043	0	0	0	0	0	0	0
b4.females_IMM	0.11172	0.14933	0.11071	0.12457	0.09858	0.1024	0.27209	0.09745	0.10638	0.10814	0.10136	0.15303	0	0	0	0	0
b4.males_IMM	0.11172	0.14933	0.11071	0.12457	0.51964	0.39262	0.3534	0.56475	0.10638	0.10814	0.10136	0.15303	0	0	0	0	0
b4.females_MILARSHI	0.4359	0.66614	0.60732	0.69537	0.09858	0.1024	0.27209	0.09745	0.10638	0.10814	0.10136	0.15303	0	0	0	0	0
b4.males_MILARSHI	0.4359	0.66614	0.60732	0.69537	0.51964	0.39262	0.3534	0.56475	0.10638	0.10814	0.10136	0.15303	0	0	0	0	0
b4.females_STOCKADE	0.24598	0.37622	0.33929	0.29059	0.09858	0.1024	0.27209	0.09745	0.10638	0.10814	0.10136	0.15303	0	0	0	0	0
b4.males_STOCKADE	0.24598	0.37622	0.33929	0.29059	0.51964	0.39262	0.3534	0.56475	0.10638	0.10814	0.10136	0.15303	0	0	0	0	0
b5.females_IMM	0.02217	0	0	0.03981	0.02013	0	0	0.02043	0.11333	0	0	0.13264	0	0	0	0	0
b5.males_IMM	0.02217	0	0	0.03981	0.16065	0	0	0.13294	0.11333	0	0	0.13264	0	0	0	0	0
b5.females_MILARSHI	0.02356	0	0	0.04959	0.02013	0	0	0.02043	0.11333	0	0	0.13264	0	0	0	0	0
b5.males_MILARSHI	0.02356	0	0	0.04959	0.16065	0	0	0.13294	0.11333	0	0	0.13264	0	0	0	0	0
b5.females_STOCKADE	0.08319	0	0	0.12526	0.02013	0	0	0.02043	0.11333	0	0	0.13264	0	0	0	0	0
b5.males_STOCKADE	0.08319	0	0	0.12526	0.16065	0	0	0.13294	0.11333	0	0	0.13264	0	0	0	0	0

Supplementary Table 4. Predicted median lifespans across capture conditions and sexes. All predictions were predicted for individuals living in the most optimal condition encountered in the sample (i.e. from the region Magway and the birth cohort 1960).

Capture status	Age at capture (yrs)	Males	Females
Captive born		30.81	44.73
Captured by stockade	5	24.84	41.18
	20	24.02	39.48
Captured by immobilisation	5	24.51	40.99
	20	23.34	38.47
Captured by milarshi	5	25.55	41.56
	20	24.03	40.37
